# Foreword from the Editor of Special Issue “Advanced Metal Chelate Complexes: Quantum-Chemical Consideration”

**DOI:** 10.3390/ma14030500

**Published:** 2021-01-21

**Authors:** Oleg V. Mikhailov

**Affiliations:** Department of Analytical Chemistry, Certification and Quality Management, Kazan National Research Technological University, K. Marx Street 68, 420015 Kazan, Russia; olegmkhlv@gmail.com

The objects of the given Special Issue of *Materials*—coordination compounds or complexes, represent a kind of “boundary zone” between two main classes of chemical substances, inorganic and organic, and therefore differ in a significantly greater variety of their structural and physicochemical characteristics in comparison with both inorganic and organic compounds. At present, these chemical compounds are objects, the reactions of their formation are the subject of a special section of chemical science called “chemistry of coordination compounds”, or, more briefly, “coordination chemistry”. In addition, although the number of currently known coordination compounds is at least a six-digit number, the concept of “coordination compound (complex)” is actually a collective one and still does not have a clear and unambiguous definition, and disputes about this have been going on almost with the emergence of coordination chemistry as such. Moreover, even the opinion of scientists as to where the stress in the keyword *complex* should be placed—on the first syllable (*cómplex*) or the second one (*compléx*)—is ambiguous. (The author of this Editorial, and at the same time, Guest Editor of the given Special Issue, adheres to the second of the above options, since the word *compléx* clearly emphasizes the individuality of the above object of chemical science, while the word *cómplex* can be associated with many concepts having a very distant relation to chemistry in general and coordination chemistry in particular, for example, “cómplex of events”, “cómplex of buildings”, etc., word combinations). However, as it is not paradoxical, the lack of a clear concept of the key object of this specific area of modern chemistry not only does not hinder its development but, moreover, allows it to integrate into its “bosom” many new objects, including even those that are difficult to fit into traditional ideas about these chemical compounds.

Coordination compounds have a number of specific (sometimes unique) properties that are not observed in those organic and/or inorganic compounds (called ligands) that are part of their composition. Moreover, the chemical properties of chemical compounds, which they possess, being ligands, often very significantly differ from the chemical properties of these compounds themselves (for example, the inclusion in the complex considerably enhances the acidic properties of both organic and inorganic compounds, which have the proton-donating ability). In this connection, even a special scientific direction—the physics-chemistry of coordinated chemical compounds (i.e., ligands)—arose in chemistry. Special interest among all currently known types of coordination compounds is the so-called metal chelate complexes (more often called “metal chelates” or even simply “chelates”) formed by polydentate organic and organo-element ligands. In this regard, it is worth noting that in accordance with the subdivision into inorganic, organic and organo-element compounds approved by the International Union of Pure and Applied Chemistry (IUPAC), organic compounds include all chemical compounds, in molecules or other structural units of which there is at least one carbon-carbon bond, and organo-element ones include those in which there is at least one carbon-carbon bond and at least one carbon-another chemical element (with the exception of hydrogen, oxygen, nitrogen, sulfur, and halogens). Hence, complexes formed by such ligands, should be classified as organic or organo-element compounds, respectively). Metal chelates differ from other coordination compounds in one specific feature: each of them contains at least one closed group of atoms, one of which is necessarily a metal atom (the so-called metal chelate ring or simply chelate ring). Within the framework of the basic systematics of metal chelates, it is customary to subdivide them into two key groups: metal chelates with the so-called open contour and metal chelates with the so-called closed contour. The first of these groups includes metal chelates containing one, two or three chelate rings; in such coordination compounds, the metal atom is on the “rim” of either a monocyclic or bicyclic system, or the so-called macrocycle that combines three chelate rings. These metal chelates usually contain the so-called compartmental ligands that are acyclic polydentate organic compounds characterized by a specific spatial orientation of donor atoms used for coordination to the corresponding metal atom. Such a specific orientation of donor atoms, however, requires a certain (usually very significant) distortion of the molecular structure of the compartmental ligand that forms the metal chelate. The second of the above groups are metal chelates, the metal atom is no longer on the “rim” of the cyclic system, but inside it in the so-called “chelate cell” formed by any so-called macrocyclic ligand (i.e., that which initially includes at least one closed grouping of at least eight atoms (the so-called macrocycle), at least four of which are donors of a lone electron pair). Unlike the metal chelates of the first group, the metal chelates of the second set contain at least four metal chelate cycles; moreover, during their formation, the molecular structure of the initial ligand forming the metal chelate either does not change at all or undergoes only minor changes in comparison with the molecular structure of the initial ligand. In this regard, such metal chelates have even received the special name “macrocyclic metal complexes” or “macrocyclic metal chelates”. Unlike the metal chelates of the first groups, the metal chelates of the second group contain at least four metal chelate rings; besides, during their formation, the molecular structure of the initial ligand forming the metal chelate either does not change at all or undergoes only minor changes in comparison with the molecular structure of the initial ligand. In this connection, such metal chelates even received the special name “macrocyclic metal complexes” or “macrocyclic metal chelates”. Note in this regard that this special issue includes articles devoted to metal chelates of both of the above groups. Other variants for the systematics of metal chelates are also possible, namely, according to the assortment of chelate rings contained in them and to the number of atoms in each of these rings; by the coordination number of the metal atom forming the metal chelate; by the nature of donor atoms that make up compartmental or macrocyclic ligand; and by the set of ligands that make up the metal chelates, etc.

One of the first (and, perhaps, the very first) metal chelates that became known to the chemical science was, apparently, a complex of nickel(II) with dimethylglyoxime (modern name is bis(dimethyldioximato)nickel(II)), obtained back in 1905 by a Russian chemist L. Chugaev as a result of the interaction of soluble nickel(II) salts with dimethylglyoxime (2,3-dimethylbutanedioxime), i.e. according to a simple scheme (metal salt + chelate ligand → metal chelate). The reaction of the formation of this substance was used for a long time later as a qualitative reaction for the detection of nickel and in quantitative (gravimetric) analysis for this element. It has not gone out of use even now, since it is nothing more than a coloring component of lipstick. For the sake of fairness, it should be noted, however, that mankind actually got acquainted with chelate and even macrocyclic compounds much earlier than the beginning of the 20th century, although they did not realize this: The fact is that the coloring components of green leaves of plants (chlorophyll) and of the blood of mammals and humans (heme) are typical metal chelates, moreover, macrocyclic chelates. Both these macrocyclic metal chelates were also subsequently obtained experimentally, but not according to the above simple scheme, but according to a much more complex scheme (metal salt + “building blocks” of the chelate ligand → metal chelate), which was called in the literature “reactions on a matrix”, “template synthesis” or “self-assembly”. The last of these terms is connected with the circumstance that at least one of the ligands that make up such a metal chelate is formed during its synthesis from simpler “building blocks”, and this self-assembly itself occurs only in the presence of certain metal ions. In the general case, self-assembly (template synthesis) is now called such processes where a metal ion (or another reaction center) with a certain stereochemistry and electronic structure acts as a pattern, template, shape or matrix for the formation of the only possible or predominant products from the corresponding initial substances’ products; besides, such a synthesis under other conditions is either extremely difficult or cannot be realized at all. Within the framework of such a synthesis, two parallel processes take place, namely the formation of a ligand system due to the “organizing” and “guiding” role of the metal ion, and the reaction of organic compounds connected with this ion (reactions of coordinated ligands). As a rule, template synthesis is used to obtain macrocyclic metal chelates (with closed contour); however, it is quite effective for the synthesis of metal chelates with an open contour, too. By the end of the XX century, the number of publications in one way or another related to the physics-chemistry of metal chelates was measured by at least a five-digit number; among them were not only original articles and reviews but also special monographs and books. In this connection, it should be noted that the overwhelming majority of compounds (both with open and closed contour) described in these works included atoms of *d*-elements and polydentate organic ligands with donor nitrogen, oxygen, and/or sulfur atoms.

All of the above applies to mononuclear metal chelates, although among the objects of modern coordination chemistry there are many polynuclear metal chelates, which are of no less (if not greater) interest than mononuclear ones. Both those and other metal chelates already found many applications in various branches of science and practice of anthropogenic activity, and the scope of their application is expanding from year to year. Among those branches of science and technology, where they are already in demand, are, in particular, the production of highly pure metals, drugs, catalysts for homogeneous and heterogeneous catalysis, and components of modern electronics and photonics. At the beginning of the XXI century, a completely new, previously unknown field of application was added to them, molecular nanotechnology. In this connection, it becomes important to calculate the structural and geometric parameters of the molecular structures of metal chelates, which determine their physicochemical properties that are somehow related to their practical use. Such a task is currently being successfully solved due to the availability of modern, already quite perfect and reliable quantum-chemical calculation methods (in particular, through the now popular Density Functional Theory (DFT)), in combination with computer software packages such as Gaussian and the corresponding computer technique. On the other hand, as a “by-product” of such calculations, the number of other parameters are determined, in particular, the distribution of the electron density and the charges associated with it on individual atoms that make up the molecular structure; the values of the relative energies of the ground and excited states; and the assessment of the stability of molecular structures, which are also important for understanding the physicochemical nature of these compounds, but which are difficult or impossible to obtain experimentally at the present level of development of science and technology. However, there are still relatively few theoretical works devoted to quantum-chemical calculations of metal chelate complexes using the DFT method (not to mention more advanced calculation methods) in the literature so far. To some extent, the reason for this lies in the great complexity of the molecular structures of most metal chelates (especially macrocyclic ones), wherefore, the quantum-chemical calculation of such compounds using even the DFT method with the simplest basis sets, in a number of cases, is very time consuming and power-consuming, and therefore very difficult for practical realization. 

Nevertheless, there is every reason to hope that in the future, with the development of computer technology, this method will become more and more available for use in scientific work, including in relation to macrocyclic compounds. In addition, it is the belief of the author that this Special Issue of *Materials* will be able to fill at least a little of this still existing gap in the theoretical and quantum chemistry of metal complexes.

